# Visual outcome after phacoemulsification with lens implant in diabetic and non-diabetic patients; A comparative study

**DOI:** 10.12669/pjms.333.12589

**Published:** 2017

**Authors:** Abdul Rashid Shaikh, Abdul Haleem Mirani, Muhammad Saleh Memon, Muhammad Faisal Fahim

**Affiliations:** 1 Abdul Rashid Shaikh, Department of Ophthalmology, Al Ibrahim Eye Hospital, Isra postgraduate Institute of Ophthalmology, Gaddap Town, Malir, Karachi, Pakistan; 2 Abdul Haleem Mirani, Department of Ophthalmology, Al Ibrahim Eye Hospital, Isra postgraduate Institute of Ophthalmology, Gaddap Town, Malir, Karachi, Pakistan; 3 Muhammad Saleh Memon, Isra Ophthalmic Research & Development Center, Al Ibrahim Eye Hospital, Isra postgraduate Institute of Ophthalmology, Gaddap Town, Malir, Karachi, Pakistan; 4 Muhammad Faisal Fahim, Isra Ophthalmic Research & Development Center, Al Ibrahim Eye Hospital, Isra postgraduate Institute of Ophthalmology, Gaddap Town, Malir, Karachi, Pakistan

**Keywords:** Phacoemulsification, Lens implant, Visual outcomes, Diabetics, Non-diabetics

## Abstract

**Objective:**

To compare the visual outcomes of phacoemulsification surgery with intraocular lens (IOL) in persons with and without diabetes at end of follow-up.

**Methods:**

This was a comparative, cross sectional, observational study with Non-probability, purposive sampling. After approval from “Research Ethical Committee of Isra Post-graduate Institute of Ophthalmology Karachi, 92 patients with cataract in one eye were selected. Patients were divided into two groups. Group A consisted of 48 diabetics and group B consisted of 44 non- diabetics with or without diabetes in the age group ≥ 30 years were included. Patients with small Pupil, Pseudo exfoliation Syndrome, Diabetic Retinopathy, and positive history of Uveitis, Glaucoma, and Macular Degeneration were excluded. Data analysis was performed by SPSS Version 20.0.

**Results:**

Best Corrected Visual Acuity (BCVA) in diabetic patients improved from 0.813 ± 0.181 Log MAR pre operatively to 0.183 ± 0.143 after the period of six months post-operatively. Corresponding results in non-diabetics were 0.66 ± 0.31 and 0.08 ± 0.092 Log Mar (P value = 0.001). If WHO criteria was considered, 87.5% diabetics and 92% non-diabetics achieved normal vision (Log Mar 0 to 0.5; ≥ 6/12,) on the first post-operative day. Remaining 12.5% diabetics and 8% non-diabetics achieved moderate vision (0.6 to 1 Log MAR) on first post-operative day improving to normal vision within a week.

**Conclusion:**

Visual outcomes in diabetics after phacoemulsification with intra ocular lens implant is almost as good as that in non-diabetic patient if the diabetics have no retinopathy and have good glycemic control.

## INTRODUCTION

Cataract is responsible for 53% of blindness in Pakistan.^1^ Diabetic patients have been found at increased risk of developing cataract when compared to persons without diabetes.^2^ With the new phenomenon of diabetes epidemic, more and more cataracts are presenting with diabetes and so more and more surgery is needed.^3^ Cataract surgery in diabetics is indicated not only to improve visual acuity, but also to allow assessment and treatment of retinopathy.^4^ Cataract surgeries are often carried out earlier amongst diabetics in developed countries to allow diagnosis and treatment of retinopathy and maculopathy.^5^ Although newer techniques have made cataract surgery safe and predictable, due to certain intrinsic problems in diabetes, visual outcome in diabetics is not as good as in non-diabetics.^6^ Diabetes are more vulnerable to intra and post-operative complications resulting in comparatively poor results. Retinopathy progresses more rapidly in diabetic patients after cataract surgery and a ruptured capsule can be a factor in rubeosis. Close follow-up and timely laser treatment are required.^7^ This study was designed to show outcome of cataract surgery by an experienced and well-trained surgeon using phacoemulsification with intra ocular lens implant in diabetic patients without diabetic retinopathy as compared to non -diabetic patients.

## METHODS

The null hypothesis for this study was “There is no difference in visual outcomes of cataract surgery in phacoemulsification with intra-ocular lens implant in persons with diabetes and without diabetes”. This was a Comparative, cross sectional, observational study. Patients were divided into two groups, first group consisted of 48 patients with diabetes (48 eyes) and the second group consisted of 44 persons without diabetes (44 eyes); Patients were examined in the outpatient department and diagnosed having Cataract in one eye. Method of Sampling was Non-probability, purposive sampling. Approval of the study was taken from “Research Ethical Committee of Isra Post-graduate Institute of Ophthalmology, Karachi. Inclusion criteria was patients attending outpatient department of AIEH from July to 2015 to December 2016, with or without diabetes in the age group ≥ 30 years, irrespective of gender, occupation and religion. Known diabetics using anti diabetic drugs were included as “diabetic” and known normal person with random sugar ≤ 140 mg/dl considered as non-diabetic. Exclusion criteria was patients with small Pupil, pseudo-exfoliation Syndrome, Diabetic Retinopathy, and positive history of Uveitis, Glaucoma, Macular Degeneration and any ocular disorder other than cataract hampering the vision.

Log MAR charts were used in this study for recording visual acuity as these charts have become gold standard method for recording visual acuity in clinical studies. Visual acuity with Snellen chart at 6 meters (6/6), at 20 feet (20/20), 1.0 vision in decimal charts and 0.0 log MAR are considered equivalent. Visual acuity was grouped into three categories as normal vision (Log MAR 0.1 to 0.5); moderate vision (0.6 to 1.0) and poor vision (≥1.1) as recommended by International Council of Ophthalmology (ICO) Sydney, Australia, April 20, 2002.^8^ On the first pre-op. visit, the patients were informed verbally about objective of the study. BCVA was taken on Log MAR Chart and recorded. Dilated Fundus examination was done and results recorded on given Performa. Diabetic patients with Hb A1C ≤ 6.5 were considered fit for surgery. All surgeries were carried out with topical anesthesia with standard phaco-emulsification procedure using Laurette Alcon machine. Injectable IOL was implanted in capsular bag. No stich was applied. No antibiotic or steroid injection was given All the surgeries were performed by one consultant.

Both study groups received post-operative topical Dexamethasone 0.1% and Moxifloxacin eye drops, eight times daily for first week. Dexamethasone drops 0.1% was continued four times daily for six weeks. All the patients were followed with a schedule of first postoperative day, one week, six weeks and six months post operatively. On each post-op visit, BCVA was checked on Log Mar and recorded in Proforma.

### Data Analysis

Data analysis was performed by SPSS Version 20.0. All the continuous variables were analyzed through mean and Standard Deviation. For categorical variables Frequency and Percentages were calculated. To know the difference between both groups with visual acuity independent sample t-test was used. P-value ≤ 0.05 considered to be statistically significant.

## RESULTS

Ninety-two patients with ninety-two eyes, 48 eyes in-group A (Diabetics) and 44 eyes in-group B (non-diabetics) were included in this study. Mean BCVA at base line in diabetics was 0.81 ± 0.18 (6/38 Snellen’s) and in- non-diabetic was 0.66 ± 0.31(6/24 Snellen’s). Six months post operatively, BCVA improved to 0.18 ±0.14 (6/7.5 Snellen’s) in-group A and to 0.08 ± 0.10 (6/6 Snellen’s) in-group B. P-value <0.001 (Table-I).

**Table-I:** Showing BCVA of Diabetic and Non-Diabetic patients.

	*Persons with diabetes*	*Persons without diabetes*	*P-value*
Pre-op BCVA	0.81±0.18 (6/38)	0.66±0.31(6/24)	-
1st Day BCVA	0.27±0.19 (6/12)	0.17±0.13 (6/7.5)	0.011
1st week BCVA	0.23±0.15 (6/9)	0.14±0.13 (6/7.5)	0.004
6th week BCVA	0.17±0.13 (6/7.5)	0.08±0.09 (6/6)	0.00
6th months BCVA	0.18±0.14 (6/9)	0.08±0.10 (6/5)	0.001

*Best Corrected Visual Acuity (BCVA)

Normal vision (Log Mar 0 to 0.5; ≥ 6/18,) was achieved by 87.5% diabetics, moderate vision (Log Mar 0.6 1 log Mar,) by 12.5% on the first post-operative day. By end of first week all, the diabetic achieved normal vision. Whereas 92% non-diabetics achieved normal vision on the first post-operative, day and 8% achieved moderate vision. Diabetics as well non-diabetics maintained normal vision by the end of the study (six months post-operative). (Table-II and III)

**Table-II:** BCVA at different follow-ups in Diabetic patients.

	*Diabetic (n=48)*			

*Vision Category Snellens = LogMar*	*Pre BCVA*	*1st day BCVA*	*6th Weeks BCVA*	*6th months BCVA*
Poor vision < 6/60 = ≥ 1.1	11 (22.9%)	0	0	0
Moderate Vision 6/24 to 6/60 = 0.6 to 1	33 (68.8%)	6 (12.5%)	0	0
Normal Vision ≥6/18 = 0 to 0.5	4 (8.3%)	42 (87.5%)	48 (100%)	48 (100%)

*Best Corrected Visual Acuity (BCVA)

**Table-III:** BCVA at different follow-ups in Non-Diabetic patients.

	*Non-Diabetics (n=44)*			

*Vision Category Snellen = Log Mar*	*Pre BCVA*	*1st day BCVA*	*6th Weeks BCVA*	*6th months BCVA*
Poor vision < 6/60 = ≥ 1.1	2 (4.5%)	0	0	0
Moderate Vision 6/24 to 6/60 = 0.6 to 1	28 (63.6%)	4 (8%)	0	0
Normal Vision ≥ 6/18 = 0 to 0.5	14 (31.8%)	40 (92%)	44 (100%)	44 (100%)

*Best Corrected Visual Acuity (BCVA)

## DISCUSSION

This study showed that 87.5% diabetic patients had normal vision (Log Mar 0 to 0.5; on Snellen ≥ 6/12) on the first post-operative day of the surgery as compared to 92% of the non-diabetics. The remaining 12.5% diabetics and 8% non-diabetics had moderate vision (0.6 to 1 Log MAR; on Snellen 6/24 to 6/60); which improved to normal vision by 1st week postoperatively.

Agha Khan University reported that, 93.3% of the operated eyes had normal vision while 4.4% and 2.2% had moderate (borderline) and poor vision respectively. Pre-existing diseases accounted for 93.9% of the moderate /poor outcome.^9^

Another study from Peshawar reported good visual outcome in 88.3%, borderline in 8.3% and poor in 3.3% patients. The main reasons for poor visual outcomes were diabetic retinopathy 42.8%, glaucoma-related vision loss 19.0%.^11^

Straatsma BR^12^ found no statistically significant difference in operative or postoperative complications in diabetics, with and without non-proliferative retinopathy, and non-diabetics in extra capsular cataract extraction with posterior chamber intraocular lens. A postoperative final visual acuity of 20/40 (6/12 Snellen 6 meters, 0.3 Log Mar) or better was achieved in 65% of diabetic eyes and in 90% of non-diabetic eyes, a difference that was statistically significant (P = 0.0049). When eyes with diabetic retinopathy or other pre-existing ophthalmic disease responsible for decreased vision were excluded, postoperative visual acuity of 20/40 or better was obtained in 93% of diabetic eyes and 96% of nondiabetic eyes, a difference that was not statistically significant (P = 0.5045).

Mittra et al.^13^ considers preoperative retinopathy and surgical inexperience an important factor in in postoperative progression of retinopathy and result irrespective of the techniques used.

Calvin Sze-un Fong^14^ reported improvement of VA by an average two lines for patients both with and without diabetes, or with DR but no past laser treatment. No improvement was evident for patients who had preoperative DR and laser therapy.

These studies show that if diabetic eye does not have retinopathy, controlled diabetes and surgery is done by an experienced surgeon, the post- operative results are comparable to non-diabetic.

This study showed good results in diabetic patients who did not have any retinopathy preoperatively, had well-controlled blood sugar level and were operated by a senior experienced surgeon.

## CONCLUSION

Visual outcome in diabetic patient after phacoemulsification with intra ocular lens implant is comparable to the results in non-diabetic patient if the diabetics have no retinopathy and have good glycemic control.
